# The relationship between the level of vitamin D, leptin and FGF23 in girls and young women with polycystic ovary syndrome

**DOI:** 10.3389/fendo.2022.1000261

**Published:** 2022-09-30

**Authors:** Agnieszka Białka-Kosiec, Dominika Orszulak, Aneta Gawlik, Agnieszka Drosdzol–Cop

**Affiliations:** ^1^ Joannitas Hospital in Pszczyna, Pszczyna, Poland; ^2^ Department of Gynaecology, Obstetrics and Oncological Gynaecology, Faculty of Health Sciences in Katowice, Medical University of Silesia, Katowice, Poland; ^3^ Department of Pediatrics and Pediatric Endocrinology, Faculty of Medical Sciences in Katowice, Medical University of Silesia, Katowice, Poland

**Keywords:** PCOS, young females, vitamin D, leptin, FGF23

## Abstract

Polycystic ovary syndrome is an endocrinopathy that mainly affects adolescent girls and young women of childbearing age. In girls, the presence of clinical and biochemical symptoms of hyperandrogenism should be particularly considered. The role of vitamin D deficiency in insulin resistance, inflammation, dyslipidemia, and obesity, i.e. in diseases associated with PCOS, has been investigated, which may suggest its involvement in the pathophysiology of the syndrome. Leptin has been shown to stimulate the formation of FGF23 in bones. There is a relationship between the incidence of dyslipidemia, adipose tissue mass and the concentration of fibroblast growth factor 23. The main aim of the presented research project is to assess the concentration of vitamin D, calcium, and selected hormones as well as the concentration of adipokines (leptin) in girls diagnosed with polycystic ovary syndrome.

**Materials and methods:**

The study included a population of 85 girls and young women aged 14 to 22 years. The study group included 37 girls who were diagnosed with polycystic ovary syndrome according to the modified Rotterdam’s criteria. The control group consisted of 48 completely healthy girls. In the first stage of the study participants were required to answer background questions. Next, anthropometric measurements were performed. The laboratory tests assessed: leptin, FGF23, FSH, SHGB, total testosterone, DHEA-S, 25-OH-D3, PTH, calcium, androstadiene, AMH, glucose, insulin.

**Results:**

The vitamin D level in the group with polycystic ovary syndrome was lower than in the control group, but there was no statistically significant difference. The level of anti-Müllerian hormone was significantly higher in the group of girls diagnosed with PCOS compared to the control group. Statistically significant differences between both groups were also noted in the HOMA-IR value. The concentration of calcium, parathyroid hormone, FGF23 and leptin in the study and control groups showed no statistically significant difference.

**Conclusions:**

In the studied group of girls with PCOS, no correlation between the level of vitamin D and selected parameters such as: AMH leptin, HOMA-IR and FGF23 was confirmed. On this basis, it can be assumed that additional vitamin D supplementation would not reduce the symptoms of polycystic ovary syndrome.

## Introduction

Polycystic ovary syndrome (PCOS) is an endocrinopathy that mainly affects adolescent girls and young women of childbearing age. The frequency of PCOS depends on the diagnostic criteria used. In girls it has not been thoroughly investigated, and in a recent study carried out on a group of women aged 15–19 it was estimated at 1.14% (1–3).

Determining the diagnosis of PCOS is difficult mainly in the group of adolescents and women in the perimenopausal period. In girls, the presence of clinical and biochemical symptoms of hyperandrogenism should be particularly considered. Some of the symptoms of polycystic ovary syndrome may be also the result of physiological changes in the body of an adolescent woman. Therefore, in this age group, the diagnosis of the syndrome is based on the modified Rotterdam’s criteria, i.e. all three Rotterdam criteria must be met (1, 4–7).

Obesity, hyperlipidemia, hyperinsulinemia, and insulin resistance are the major metabolic disorders accompanying PCOS. Hyperinsulinemia and insulin resistance occur in 20-40% of slim women with PCOS and in as many as 80% of obese patients. The coexistence of polycystic ovary syndrome and obesity shows a synergistic, unfavorable effect on the insulin. In adolescents, ovarian volume is correlated with levels of free testosterone and insulin, as well as with insulin resistance. Insulin also stimulates hyperkeratosis and the proliferation of skin fibroblasts. Thus, in PCOS patients, acanthosis nigricans can be observed more often (1, 2, 8).

The pleiotropic effect of vitamin D is evidenced by the fact that VDR (Vitamin D Receptor) is present in cells of various tissues, not only those related to calcium and phosphorus homeostasis, but also in 1-alpha hydroxylase (the enzyme necessary for the formation of vitamin’s active form in kidneys) which can be found in keratocytes, macrophages, enterocytes or cells of the human placenta. VDR regulates the expression of approximately 3% of human genetic material (9–12).

Vitamin D has been shown to work in the uterus, ovary, vagina, and placenta. It affects the implantation of the embryo, the course of pregnancy and the health of the progeny. It must be acknowledged that the evidence supporting its contribution to human reproductive health is inconsistent and based mainly on observational studies. The role of vitamin D deficiency in insulin resistance, inflammation, dyslipidemia, and obesity, i.e. in diseases associated with PCOS, has been investigated which may suggest its involvement in the pathophysiology of the syndrome. In their studies, Keshavarz et al. found vitamin D deficiency <20ng/ml in 78.7% of patients with PCO syndrome, and its normal level was only in 2.7% of patients (5, 12, 13)

Fibroblast growth factor 23 (FGF23) is a part of newly identified regulators system that forms the FGF23-bone-kidney axis, with a function comparable to the PTH-vitamin D axis. It is mainly synthesized by osteocytes and osteoblasts, qualifying bone tissue as an endocrine organ (14, 15).

The kidney is the target organ of FGF23 and the 1,25(OH)_2_D_3_ is its main regulating factor. Excessive secretion of fibroblast growth factor 23 leads to hypophosphatemia, a decrease in 1,25(OH)_2_D_3_ concentration and to rachitis or osteomalacia. In turn, FGF23 deficiency results in hyperphosphatemia, an increase in the 1,25(OH)_2_D_3_ concentration and the formation of calcifications in soft tissues. The cofactor of FGF23 is the Klotho protein. The PTH, by stimulating the activity of 1α-hydroxylase, increases the synthesis of 1,25(OH)_2_D_3_, which in turn enhances the synthesis of the Klotho protein (14, 15).

Leptin has been shown to stimulate the formation of FGF23 in bones. There is a relationship between the incidence of dyslipidemia, adipose tissue mass and the concentration of fibroblast growth factor 23. It has also been shown that estrogens, deficiency of which is the main risk factor for osteoporosis, stimulate the secretion of FGF23 (14, 15).

Changes in the secretion of adipokines are an important link in the development of insulin resistance in peripheral tissues. In this mechanism adipokines may indirectly participate in the pathogenesis of PCO syndrome. Earlier studies showed that the concentration of leptin is significantly higher in PCOS patients who have irregular menstrual cycles compared to the group with regular cycles. The level of fertility in women with PCOS has been shown to be inversely related to the concentration of circulating leptin. In turn, in adolescents with polycystic ovary syndrome, leptin concentration depends on BMI, adipose tissue content, waist circumference and HOMA-IR value (16, 17).

So far, the published literature data assess the level of vitamin D, without explanation of the changes taking place, etiology, and pathogenesis of PCOS. The possible relationship between fibroblast growth factor 23 and leptin in the pathogenesis of PCOS has not been investigated yet. The conducted studies focused almost exclusively on the group of adult patients. The results of the studies published so far are not unequivocal.

The main aim of the presented research project is to assess the concentration of vitamin D, calcium, and selected hormones as well as the concentration of adipokines (leptin) in girls diagnosed with polycystic ovary syndrome. The first symptoms of PCOS, such as hyperandrogenism and anovulatory cycles, usually appear in adolescence, but the moment when the patient visits the doctor, and the diagnosis is made is most often delayed. In this situation, conducting research is particularly important not only for the development of diagnostics and treatment, but also for examining the pathophysiology of the syndrome. Years later, the patients show such multi-system symptoms that it is difficult to recognize the initiating factors.

The possible relationship between fibroblast growth factor 23 and leptin in the pathogenesis of PCOS has not been investigated so far.

The specific aims are:

1. Assessment of the variability of vitamin D levels depending on the occurrence of the syndrome polycystic ovaries.
2. Assessment of the variability of AMH concentrations depending on the presence of polycystic syndrome ovaries.
3. Determination of the relationship between AMH, FGF23, leptin, HOMA-IR and the level vitamin D in the test and control group.

## Materials and methods

### Materials

The study included a population of 85 girls and young women aged 14 to 22 years. Patients were recruited for the study at the Department of Pediatrics and Pediatric Endocrinology of the John Paul II Upper Silesian Child Health Centre in Katowice and at the children’s gynecology sub-department in the Department of Gynaecology, Obstetrics and Oncological Gynaecology in Katowice. The study was conducted between December and March.

The study was conducted between December and March. The study group included 37 girls who were at least two years after the menarche and were diagnosed with polycystic ovary syndrome according to the modified Roterdam’s criteria. The mean length of menstrual cycles in the study group was 59.03 days (± 45.17 days). The values of the performed hormonal tests (androstenedione, testosterone, DHEAS and FAI) were increased and on their basis the hyperandrogenism was diagnosed. The exact inclusion criteria for the study group are summarized in **Table 1**.

**Table 1:** The inclusion and exclusion criteria for the study group.

Inclusion Criteria:	Exclusion Criteria:
-age between 14 and 22 years	-pharmacotherapy used in the last 6 months (including hormonal drugs, contraceptives, NSAIDs)
-time over 2 years from the menarche	-additional systemic diseases (e.g., cardiovascular diseases, diabetes, gastric/duodenal ulcer disease, autoimmune diseases)
-consent of a woman or a girl and her legal guardian to participate in the study	-use of dietary supplements, especially those containing vitamin D or calcium, within the last 12 months
-oligo- or anovulation	-sunbathing in the last 3 months
-biochemical indicators of hyperandrogenism or hirsutism	-applying a restrictive diet in the last 12 months
-volume of one of the ovaries >12 ml or >24 follicles in the ovary in pelvic ultrasound examination	- endocrinopathies (congenital adrenal hyperplasia, Cushing’s syndrome, hyperprolactinemia, thyroid dysfunction, acromegaly, androgen-secreting tumors)

The study group was recruited from the patients’ population of the hospital outpatient clinic at Department of Pediatrics and Pediatric Endocrinology of the John Paul II Upper Silesian Child Health Centre in Katowice and the Department of Gynaecology, Obstetrics and Oncological Gynaecology in Katowice, during follow-up visits. The control group consisted of 48 completely healthy girls aged between 14 and 22 years old, at least two years after the menarche, menstruating regularly (28 ± 7 days) for at last six months. In the control group the presence of hyperandrogenism was not confirmed in laboratory tests. The exclusion criteria for this group remained the same as for the study group (**Table 1**).

All participants of the study were informed in detail about its purpose and method. The consent to take part in the study was obtained from all participants (in case of patients under 18 years of age the consent was obtained from both the respondent and her parents/legal guardians). The consent of the Bioethical Committee of the Medical University of Silesia in Katowice was obtained to conduct the study (consent number - KNW/0022/KB1/136/III/13/14/16).

### Methodology

In the first stage of the study participants were asked to answer background questions about medical history (regarding internal diseases, use of dietary supplements, exposure to the sun, addictions) and gynecological history with the assessment of the menstrual cycle.

The normal menstrual cycle in girls was defined as:

- cycles lasting 21-45 days;
- menstrual bleeding 3-7 days;
- blood loss during menstruation 5-80 ml.

Secondary amenorrhea was diagnosed in the event of a lack of period for six months after the previous period of normal menstruation.

In the next stage the anthropometric measurements (height, weight, BMI) were performed. The severity of hirsutism was assessed according to the Ferriman-Gallwey score (diagnosed hirsutism ≥ 8 points). The ultrasound examination of the pelvis was performed. The laboratory tests assessed: leptin, FGF23, FSH, SHGB, total testosterone, DHEA-S, 25-OH-D_3_, PTH, calcium, androstadiene, AMH, glucose, insulin. The characteristics of the studied biochemical parameters are presented in *Additional data section*.

Insulin resistance was assessed indirectly based on the obtained results after calculating the value of the HOMA-IR indicator. The formula was as follows: HOMA-IR (Homeostasis Model Assessment) = fasting serum insulin concentration (mU/ml) x fasting serum glucose concentration (mmol/L)/22.5.

Insulin resistance was diagnosed at HOMA-IR values ≥ 2.5.

Free Androgen Index (FAI) was calculated using the following formula: FAI = [Total Testosteron/SHBG] x 100%. Normal values <5%.

Laboratory research was made in the Biochemical Laboratory of the Department of Health Promotion and Obesity Management, Department of Pathophysiology, Faculty of Medical Sciences, Medical University of Silesia in Katowice.

### Statistical analysis

The Excel 2007 and STATISTICA v.12PL applications were used for the statistical analysis. The result of the statistical analysis was considered statistically significant if the obtained significance level “p” was less than or equal to 0.05.

The statistical research used:

- Shapiro-Wolf test;
- Mann-Whitney U test;
- CHI2 test with Yates correction;
- Spearman and Kendall correlation test;
- analysis of discrimination - a one-time, multi-parameter comparison of two groups.

## Results

### General characteristics of the studied groups of girls

The mean age of the participants from the study group was 19.4 ± 2.4 years, while in the control group it was 20.0 ± 2.2 years. The difference between the two groups in this parameter was not statistically significant.

There were no statistically significant differences between the two groups also in terms of body weight, height, and body mass index (BMI). The above data are presented in **Table 2**.

**Table 2:** Basic characteristics of anthropometric data in groups.

Statistical parameter	Study group		Control group		The Mann-Whitney U Test
	[mean value ± SD]	Median	[mean value ± SD]	Median	
Height [cm]	165,9 ± 6,5	167	165,5 ± 6,1	165,0	p=0,49
Body weight [kg]	64,3 ± 12,4	62,5	61,8 ± 9,9	60,0	p=0,31
BMI [kg/m2]	23,3 ± 4,1	22,6	22,6 ± 3,5	22,3	p=0,55

### Comparison of biochemical parameters in the test and control group

The vitamin D level in the group with polycystic ovary syndrome was lower than in the control group (**Table 3**). The mean concentration in the study group was 24.88 ng/ml. However, no statistically significant difference was found in these measurements (p=0.80). In both groups the mean level of vitamin D was below the reference value.

**Table 3:** Vitamin D3, AMH, HOMA-IR level in the study and control group.

Parameter	Group	Group size	Mean	Standard deviation	Median	Min value	Max value	Normality test	The Mann-Whitney U Test
Vitamin D	Study	37	24,88	11,54	22,12	9,31	52,64	p=0,02	NS (p=0,80)
	Control	48	26,29	14,15	22,63	4,62	69,26	p=0,01	
AMH	Study	35	9,21	4,57	8,16	1,84	20,37	p=0,18	p=0,001
	Control	48	6,25	3,67	5,31	1,05	17,19	p=0,002	
HOMA-IR	Study	33	2,62	1,92	2,34	0,58	11,04	p<0,000001	p=0,003
	Control	48	1,80	1,82	1,47	0,13	12,39	p<0,000001	

The level of anti-Müllerian hormone was significantly higher in the group of girls diagnosed with PCOS compared to the control group (p=0.001). The mean concentration of AMH in the study group was 9.21 ng/ml. A relationship between the level of the anti-Müllerian hormone and the occurrence of polycystic ovary syndrome was demonstrated. Statistically significant differences between both groups were also noted in the HOMA-IR value (2.62 ± 1.92 in the study group and 1.80 ± 1.82 in the control group; p=0.003) (**Table 3**).

No statistical significance between the control group and the study group in terms of the concentration of calcium, parathyroid hormone, FGF23 and leptin was noticed. The results are detailed in **Table 4**.

**Table 4:** Ca, PTH, FGF23, leptin values in the study and control group.

Parameter	Group	Group size	Mean	Standard deviation	Median	Min value	Max value	Normality test	The Mann-Whitney U Test
FGF23	Study	30	59,67	29,95	52,59	21,86	168,19	p=0,001	NS (p=0,99)
	Control	48	58,57	26,20	52,19	23,25	168,25	p<0,000001	
Ca	Study	30	2,25	0,13	2,26	1,75	2,48	p=0,0002	NS (p=0,45)
	Control	48	2,22	0,18	2,25	1,46	2,47	p=<0,000001	
PTH	Study	30	19,10	7,81	17,68	5,07	46,21	p=0,009	NS (p=0,59
	Control	48	19,46	9,68	19,67	3,51	38,20	p=0,14	
Leptin	Study	37	15,32	10,10	12,07	2,92	48,56	p=0,001	NS (p=0,15)
	Control	48	13,86	13,56	9,82	2,68	80,35	p<0,000001	

### Correlations of selected parameters in the test and control group

**Figure 1:**
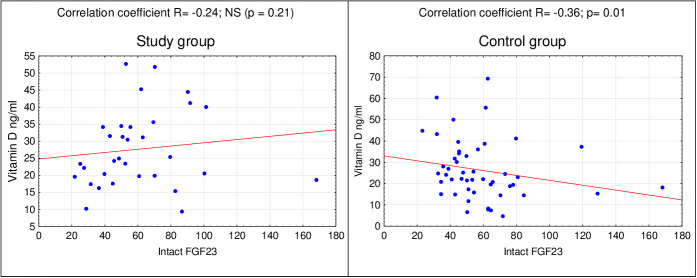
Correlation of FGF23 and vitamin D in the study and control group.

In the next stage of statistical analyzes, relationships between the concentrations of selected markers in the study group and the control group were assessed. In the control group, using the Spearman’s correlation, a relationship between the concentration of vitamin D and the concentration of FGF23 (p=0.01) (**Figure 1**) was found. There was no correlation between:

- vitamin D and leptin levels;
- vitamin D and AMH levels;
- leptin and FGF23 concentration (**Table 5**).

**Table 5:** Spearman’s correlations in the study and control group.

Correlation	Study group	Control group
Vitamin D - FGF23	R=0,24; NS (p=0,21)	**R=-0,36; p=0,01**
FGF23 - Leptin	R=-0,18; NS (p=0,34)	R=0,11; NS (p=0,47)
Vitamin D - Leptin	R=-0,05; NS (p=0,75)	R=0,05; NS (p=0,73)
Vitamin D - AMH	R=0,06; NS (p=074)	R=0,11; NS (p=0,44)
Leptin - FGF23	R=-0,18; NS (p=0,34)	R=0,11; NS(p=0,47)
HOMA-IR - AMH	R=-0,08; NS (p=0,65)	R=0,03; NS (p=0,87)
HOMA-IR - BMI	R=0,22; NS (p=0,22)	**R=0,32; p=0,03**
AMH - BMI	R=-0,15; NS (p=0,38)	R=0,10; NS (p=0,49)

## Discussion

According to previously published studies, the serum levels of vitamin D in women with PCO syndrome may be higher, lower, or not significantly different than in healthy controls. This leads to the conclusion that the role of vitamin D in the pathogenesis of PCOS is unclear (18). In the conducted study, lower levels of vitamin D were found in patients with PCOS, but the difference was not statistically significant.

In the study by Ghadimi et al. (19) the mean values ​​of vitamin D were significantly lower in the group of girls (192 girls aged 16-20) with the PCO syndrome in comparison to the healthy control group. Similarly, to the present study they did not show any difference in the level of calcium between the groups, even though animal studies have proven its effect on oocyte maturation and impairment of its regulation system on follicle arrest (8). There was also no correlation between the severity of vitamin D deficiency and the severity of acne, hirsutism, and obesity. Moreover, there was no correlation between the level of vitamin D and the lipid and hormonal profile of both groups (19). On the other hand, in a British study on a group of adult women, no significant difference between the level of vitamin D in patients with and without PCOS was found, although severe deficiency was more common in the study group. However, this study showed an inverse correlation between BMI and serum 25 (OH) D concentration in women with PCOS (18).

While reviewing the literature, one can meet the hypothesis that the level of vitamin D correlates with the BMI, so there is a relationship between the level of vitamin and obesity, and it is not dependent on the occurrence of PCOS (19). However, it should be considered that obese people spend less time outdoors when exposed to sunlight, which leads to insufficient vitamin D biosynthesis in the skin and is the main reason for the difference in 25 (OH) D levels in this group. In the conducted studies, it was noticed that in obese subjects 24 hours after exposure to UV light, the increase in 25 (OH) D level was by 57% lower than in healthy subjects. This may be due to the reduction of vitamin D bioavailability through its sequestration in excessive amounts of adipose tissue. Of course, food preferences should also be considered, as well as vitamin D metabolism in individual groups and their possible impact on the results of the analyzes (18). On the other hand, it has been suggested that the relationship between vitamin D levels and FAI levels results from the reduction of SHBG because of obesity (insulin inhibits SHBG synthesis in the liver), which would support the hypothesis that D hypovitaminosis is only the result of obesity in PCO syndrome (20). Tsakova et al. (21) showed that obese women with PCOS have lower 25 (OH) D levels compared to slim PCOS patients.

In the study by Keshavarz et al. no relationship between the concentration of vitamin D and total testosterone, DHEAS, SHBG and the index of free androgens was found (13). Similarly, in the American study from 2016, no relationship between the level of vitamin D and the concentration of total testosterone, FAI, and hirsutism was found (5).

**Figure 2:**
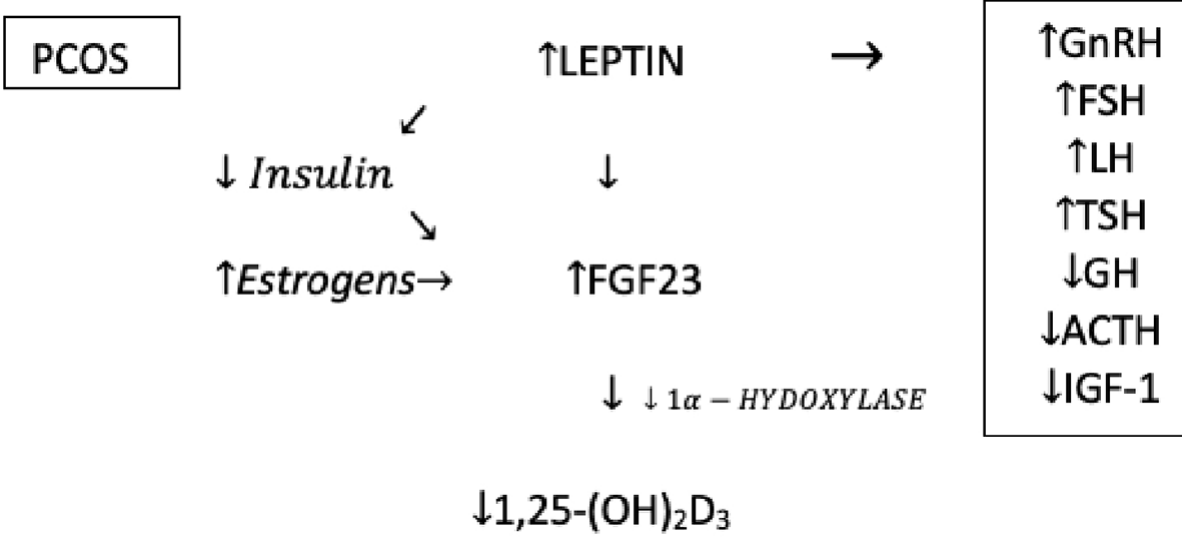
Potential relationship between leptin, FGF23 and vitamin D levels in patients with PCOS.

Analyzing the articles about polycystic ovary syndrome, it was noticed that some seemingly unrelated elements could form a causal relationship and be involved in etiopathogenesis and explain some of the symptoms of PCOS. FGF23 turned out to be the central element in this hypothesis. Its formation is stimulated by leptin and estrogens, and FGF23 itself reduces the concentration of 1,25(OH)_2_D_3_ reducing thereby the activity of 1α-hydroxylase (**Figure 2**) (14, 15).

Despite attempts to fully understand the mechanisms that regulate the FGF23 production and secretion it should be unequivocally stated that this is an extremely complex and complicated issue that has not been fully described yet. Leptin has a major influence on the increased level of FGF23. It was proven that the secretion of FGF23 in the osteocytes is directly stimulated by leptin and can have an important impact on several mineral metabolism parameters. Furthermore, as with FGF23, leptin also inhibits the synthesis of 1,25(OH)2D3 (22).

Another hormone that has a major impact on the bone metabolism by regulating bone markers including FGF23, vitamin D, DBP, especially in women, is estrogen. Estrogen therapy can decrease the FGF23 level, due to the phosphaturia effect of estrogen. Treatment with exogenous estradiol leads to an increase in 25(OH)D3, which increases the active form 1,25(OH)2D3 in women (22).

Baldani et al. found higher levels of leptin in PCOS patients compared to healthy controls. The same relationship was obtained after the groups were verified in terms of BMI, which suggests that the relationship is not only the result of higher body weight of patients in the study group. However, they did not notice any correlation between the level of leptin and the concentration of total testosterone and HOMA-IR in the analyzed groups. It was suggested that obesity and the diagnosis of PCOS itself affect the serum leptin level almost equally (23). In our study, we did not find a statistically significant difference in the level of leptin between the control group and the study group. According to other publications, in adolescent patients the concentration of leptin was correlated with the HOMA-IR value (16, 17).

It was noted that in rodents, insulin deficiency was an independent factor increasing the serum levels of FGF23. The administration of insulin reversed this effect. Also, a negative correlation between FGF23 and insulin was observed in humans (24). In our research, we noticed a relationship between the concentration of vitamin D and FGF23, but only in the control group. There was also no correlation between FGF23 and leptin or vitamin D and leptin in any of the groups.

In the studied group of girls with PCOS, the existence of a relationship between the level of vitamin D and selected parameters such as leptin and FGF23 was not confirmed. On this basis, it can be assumed that additional vitamin D supplementation would not reduce the symptoms of polycystic ovary syndrome. On the other hand, in the control group there was a correlation between the vitamin D concentration and the concentration of FGF23. However, the presented relationship is not clinically significant. Further studies of these relationships in a larger group of patients are necessary.

The results of this study do not explain the pathogenesis of PCOS but are another element of research aimed at trying to determine the processes that affect the appearance of the clinical picture presented by patients. The presented results show that the average vitamin D concentration in the study and control group is below the reference value. Moreover, it is suggested that girls diagnosed with PCOS have higher levels of AMH and HOMA-IR. Considering the declining diagnostic value of ultrasound scan in girls in PCOS diagnosis, further studies leading to a discovery of new markers enabling an early diagnosis of the disease are definitely needed.

## Limitations of the study

The main limitation of the study is the small number of participants in both study and control groups. This is due to the limited ability to qualify girls, caused by the infrequent prevalence and diagnosis of polycystic ovary syndrome in this age group. The use of HOMA-IR for the assessment of insulin resistance, i.e. an indirect method instead of the metabolic clamp being the “gold standard”, should also be considered as a limitation of the study. Such a choice was made due to the safety of the adolescent patient and the ambulatory conditions in which the tests were carried out. Moreover, the reference value for the HOMA-IR and AMH index (marked in the conducted study) in the group of adolescent patients has not been established so far.

## Additional data section

The characteristics of the studied biochemical parameters:

1. Androstendion
Method sensitivity: 0.40 ng/ml
The coefficient of variation within and between laboratories was 5.2%, 8.7%,
Normal: 0.7-3.1ng/ml
2. AMH (Anti-Mullerian Hormone)
Method sensitivity: 0.08 mg/ml
The coefficient of variation within and between laboratories is as follows: 6.75%, 5.5%
Normal: 0-10.6 ng/mL
3. FSH
Method sensitivity: 0.1 mIU/ml
The coefficient of variation within and between laboratories was 1.7%, 1.7%
Normal: 3.5-12.5 mlU/ml
4. SHBG
Method sensitivity: 0.35 nmol/l
The coefficient of variation within and between laboratories was: 2.4%, 2.2%,
Norm: 26.1 -110 nmol/l
5. Total testosterone
Method sensitivity: 0.0250 nmol/L
The coefficient of variation within and between laboratories was 2.5%, 1.6%
Norm: 0.290-1.67 nmol/l
6. DHEA-S
Method sensitivity: 0.1 μg/dL
43
The coefficients of variation within and between laboratories were 2.3%, 2.5%
Normal: 65.1 - 368 μg/dL
7. Insulin
Method sensitivity: 0.2 μIU/ml
The coefficient of variation within and between laboratories was 1.9%, 1.6%
Normal: 2.6 - 20 μIU/ml
8. Parathyroid hormone
Method sensitivity: 1.20 pg/mL, 0.127 pmol/L
The coefficient of variation within and between laboratories was 1.6% and 2.2%
Standard: 15-65pg/mL, 1.6-6.9 pmol/L
9. 25- Hydroxyvitamin D
Method sensitivity: 9.0 ng/mL
The coefficient of variation within and between laboratories was 5.1% and 2.7%
Normal:> 30 ng/mL
10. Calcium
Method sensitivity: 0.20mmol/L, 0.8 mg/dL
The coefficient of variation within and between laboratories was 1.9% and 1.9%
Normal: 2.10-2.55 mmol/L, 8.4-1.2 mg/dL
11. Leptin
Method sensitivity: 0.2 ng/ml,
The coefficient of variation within and between laboratories was 5.9%, 5.5%
12. FGF23
Method sensitivity: 0.04pg/mL
The coefficients of variation within and between laboratories were: 8.9% and 12.4%

## Data availability statement

The raw data supporting the conclusions of this article will be made available by the authors, without undue reservation.

## Ethics statement

The studies involving human participants were reviewed and approved by Ethic Committee of Medical University of Silesia Katowice 40-055 ul. Poniatowskiego 15. Written informed consent to participate in this study was provided by the participants’ legal guardian/next of kin. Written informed consent was not obtained from the individual(s) for the publication of any potentially identifiable images or data included in this article.

## Author contributions

AB-K - study conception and design, analysis and interpretation of data, drafting the article, revising the article critically for important intellectual content. DO - analysis and interpretation of data, revising the article critically for important intellectual content. AG **-** study conception and design, revising the article critically for important intellectual content, final approval of the version to be published. AD-C - study conception and design, analysis and interpretation of data, drafting the article, revising the article critically for important intellectual content, final approval of the version to be published. All authors contributed to the article and approved the submitted version.

## Funding

This study was funded by Medical University of Silesia in Katowice.

## Acknowledgments

The Authors wish to thank Andrzej Widera for his support in statistical processing of the results and Michał Skorupa- Kopczyński for proofreading of the manuscript.

## Conflict of interest

The authors declare that the research was conducted in the absence of any commercial or financial relationships that could be construed as a potential conflict of interest.

## Publisher’s note

All claims expressed in this article are solely those of the authors and do not necessarily represent those of their affiliated organizations, or those of the publisher, the editors and the reviewers. Any product that may be evaluated in this article, or claim that may be made by its manufacturer, is not guaranteed or endorsed by the publisher.
